# Expanding the genotype and phenotype spectrum of *SYT1*-associated neurodevelopmental disorder

**DOI:** 10.1016/j.gim.2021.12.002

**Published:** 2022-04

**Authors:** Holly Melland, Fabian Bumbak, Anna Kolesnik-Taylor, Elise Ng-Cordell, Abinayah John, Panayiotis Constantinou, Shelagh Joss, Martin Larsen, Christina Fagerberg, Lone Walentin Laulund, Jenny Thies, Frances Emslie, Marjolein Willemsen, Tjitske Kleefstra, Rolf Pfundt, Rebekah Barrick, Richard Chang, Lucy Loong, Majid Alfadhel, Jasper van der Smagt, Mathilde Nizon, Manju A. Kurian, Daniel J. Scott, Joshua J. Ziarek, Sarah L. Gordon, Kate Baker

**Affiliations:** 1 The Florey Institute of Neuroscience and Mental Health, University of Melbourne, Parkville, Victoria, Australia; 2 Melbourne Dementia Research Centre, The Florey Institute of Neuroscience and Mental Health, University of Melbourne, Parkville, Victoria, Australia; 3 Department of Molecular and Cellular Biochemistry, College of Arts + Sciences, Indiana University Bloomington, Bloomington, IN; 4 MRC Cognition and Brain Sciences Unit, University of Cambridge, Cambridge, United Kingdom; 5 Department of Clinical Genetics, Queen Elizabeth University Hospital, Glasgow, United Kingdom; 6 Department of Clinical Genetics, Odense University Hospital, Odense, Denmark; 7 Hans Christian Andersen Children’s Hospital, Odense University Hospital, Odense, Denmark; 8 Department of Pediatrics, Division of Genetic Medicine, Seattle Children’s Hospital, Seattle, WA; 9 South West Thames Regional Genetics Service and St George's University of London, London, United Kingdom; 10 Radboud University Medical Center, Nijmegen, The Netherlands; 11 Vincent van Gogh Centre for Neuropsychiatry, Venray, The Netherlands; 12 Children's Hospital of Orange County, Orange, CA; 13 Oxford Centre for Genomic Medicine, Oxford University Hospitals NHS Foundation Trust, Oxford, United Kingdom; 14 Genetics and Precision Medicine department, King Abdullah Specialized Children Hospital, King Abdulaziz Medical City, Ministry of National Guard Health Affairs, Riyadh, Saudi Arabia; 15 Medical Genomics Research Department, King Abdullah International Medical Research Center, Ministry of National Guard Health Affairs, Riyadh, Saudi Arabia; 16 College of Medicine, King Saud bin Abdulaziz University for Health Sciences, King Abdulaziz Medical City, Ministry of National Guard Health Affairs, Riyadh, Saudi Arabia; 17 Utrecht University Medical Centre, Utrecht, The Netherlands; 18 Service de Génétique Médicale, CHU de Nantes, INSERM, Université de Nantes, Nantes, France; 19 Developmental Neurosciences Programme, UCL Great Ormond Street Institute of Child Health, University College London, London, United Kingdom; 20 Department of Medical Genetics, University of Cambridge, Cambridge, United Kingdom

**Keywords:** Intellectual disability, Neurotransmission, Synapse, Synaptic vesicle, Synaptotagmin

## Abstract

**Purpose:**

Synaptotagmin-1 (SYT1) is a critical mediator of neurotransmitter release in the central nervous system. Previously reported missense *SYT1* variants in the C2B domain are associated with severe intellectual disability, movement disorders, behavioral disturbances, and electroencephalogram abnormalities. In this study, we expand the genotypes and phenotypes and identify discriminating features of this disorder.

**Methods:**

We describe 22 individuals with 15 de novo missense *SYT1* variants. The evidence for pathogenicity is discussed, including the American College of Medical Genetics and Genomics/Association for Molecular Pathology criteria, known structure–function relationships, and molecular dynamics simulations. Quantitative behavioral data for 14 cases were compared with other monogenic neurodevelopmental disorders.

**Results:**

Four variants were located in the C2A domain with the remainder in the C2B domain. We classified 6 variants as pathogenic, 4 as likely pathogenic, and 5 as variants of uncertain significance. Prevalent clinical phenotypes included delayed developmental milestones, abnormal eye physiology, movement disorders, and sleep disturbances. Discriminating behavioral characteristics were severity of motor and communication impairment, presence of motor stereotypies, and mood instability.

**Conclusion:**

Neurodevelopmental disorder–associated *SYT1* variants extend beyond previously reported regions, and the phenotypic spectrum encompasses a broader range of severities than initially reported. This study guides the diagnosis and molecular understanding of this rare neurodevelopmental disorder and highlights a key role for SYT1 function in emotional regulation, motor control, and emergent cognitive function.

## Introduction

The tightly regulated synaptic vesicle cycle involves the trafficking, docking, fusion, and recycling of neurotransmitter-filled vesicles at the presynaptic terminal. Precision and efficiency of these processes is critical for synchronous neurotransmission, neural network development, and emergent cognitive functions.^1^^,^^2^ Inherited and de novo variants in more than 40 different synaptic vesicle cycling genes have been associated with a broad spectrum of neurodevelopmental phenotypes, including epilepsies, movement disorders, delayed acquisition of motor milestones, intellectual disability (ID), visual impairment, and emotional-behavioral disturbances.^3^ Improving the delineation, diagnosis, and management of these disorders requires comprehensive phenotyping in parallel with detailed genetic, molecular, and cellular analysis of variants.

Variants in the *SYT1* gene, which codes for the protein synaptotagmin-1 (SYT1), give rise to *SYT1*-associated neurodevelopmental disorder, also known as Baker-Gordon Syndrome (OMIM 618218). SYT1 is a synaptic vesicle protein that couples action potentials to the synchronous exocytosis of neurotransmitters through its calcium sensing activity.^4^ SYT1, the dominant synaptotagmin family member in the forebrain,^5^^,^^6^ is a transmembrane protein with 2 cytoplasmic calcium-binding domains (C2A and C2B).^7^ Membrane depolarization triggers an influx of calcium ions (Ca^2+^) into the nerve terminal, which bind to negatively charged aspartate residues that reside in loops at the “top” of each C2 domain. This neutralizes the charge of the loops, acting as an electrostatic switch and allowing the hydrophobic tips of these loops to penetrate the negatively-charged plasma membrane, thereby facilitating the fusion of synaptic vesicle and plasma membranes.^4^

We previously reported 11 cases of de novo missense variants in *SYT1*.^8^^,^^9^ All affected individuals presented with hypotonia, developmental delay, and ID varying in severity from moderate to profound and one-third exhibited symptoms of an involuntary movement disorder. Behavioral characteristics included unpredictable switches from placidity to agitation and pronounced motor stereotypies such as hand-biting. Electroencephalograms (EEGs) were abnormal in all cases, characterized by intermittent low-frequency, high-amplitude oscillations. Five different *SYT1* variants were identified (Met303Lys, Asp304Gly, Asp366Glu, Ile368Thr, and Asn371Lys), all located in highly conserved residues of the C2B domain of SYT1 that cluster around the Ca^2+^-binding pocket. These missense variants were found to inhibit evoked exocytosis in a dominant-negative and variant-specific manner.^9^^,^^10^

The current paper expands on the genotypic and phenotypic spectrum of *SYT1*-associated neurodevelopmental disorder. We evaluate evidence for pathogenicity of novel variants through in silico analysis and molecular dynamics simulations. We provide a quantitative evaluation of behavioral characteristics within the cohort and a comparison with individuals with other monogenic neurodevelopmental disorders.

## Materials and Methods

### Recruitment and sample description

*SYT1* variants were identified via exome sequencing (trio or solo) within clinical laboratories or ethically approved research studies. The identification, validation, confirmation of de novo status, and clinical reporting of *SYT1* variants were carried out by each participant’s clinical center. Authors were notified of diagnosed variants by personal communication, through database searching of ClinVar^11^ (https://www.ncbi.nlm.nih.gov/clinvar/) or Decipher^12^ (https://www.deciphergenomics.org/), or through GeneMatcher^13^ (https://genematcher.org/statistics/). A total of 51 additional participants with ID of known monogenic origin (excluding synaptic vesicle cycling disorders as listed by John et al^3^) were recruited as a comparison group for behavioral data. Genetic diagnoses within the comparison group are listed in Supplemental Table 1.

### Evaluation of variants

Evaluation of pathogenicity for the 15 de novo sequence variants followed the American College of Medical Genetics and Genomics/Association for Molecular Pathology (ACMG/AMP) classification guidelines^14^ supplemented by the Association for Clinical Genomic Science United Kingdom best practice guidelines^15^ (for details see Supplemental Methods).

### Molecular dynamics simulations

Homology models of SYT1 C2 domains harboring variants were generated from solution structures of the Ca^2+^-bound C2A and C2B domains of rat SYT1 (Protein Data Bank: 1BYN^16^ and 1K5W^17^). Note that amino acid numbering used throughout this paper follows human sequence numbering for simplicity. The wild-type (WT) structure and each variant homology model (with Ca^2+^ ions both present and removed) were subjected to 4 individual atomistic molecular dynamics simulations with trajectory lengths of approximately 400 ns each (for full details see Supplemental Methods). Root-mean-square deviation of the backbone and root-mean-square fluctuations (RMSF) of the backbone C-alpha atoms of each domain variant were measured over the course of the simulations as readouts of overall and local mobility of the domains. To assess Ca^2+^ mobility and binding pocket occupancy, the distance between each Ca^2+^ atom and a reference amino acid was measured over time.

### Phenotyping methods and analysis

Clinical information for all individuals with de novo *SYT1* variants was collated from clinical documentation and parent-report questionnaires (online or by post) using a standard template (for individual clinical histories see Supplemental Table 2). The study-specific Medical History Interview gathered information about perinatal history, infant and child health, neurological symptoms, and developmental milestones. The Vineland Adaptive Behavior Scales is a standardized assessment tool for everyday adaptive functioning commonly used to support evaluation of neurodevelopmental disorders. Within the sample, either second edition^18^ (ID control, *n* = 34) or third edition^19^ (SYT1, *n* = 14; ID control, *n* = 17) of the Vineland Behavior Scales was used. The Developmental Behavior Checklist 2^20^ (DBC-P) assesses emotional and behavioral problems in individuals with ID and comprises 5 subscales (disruptive/antisocial, self-absorbed, communication disturbance, anxiety, and social relating). The Social Responsiveness Scale^21^ is a standardized questionnaire enquiring about the presence and severity of social impairments (social motivation, social awareness, social cognition, social communication, and restricted interests and repetitive behavior). Statistical analysis was performed using SPSS Statistics version 27 (IBM), and the distributions of all outcome measures were examined for normality before parametric or nonparametric analyses, as appropriate.

## Results

### *SYT1* Variants

**Figure 1:**
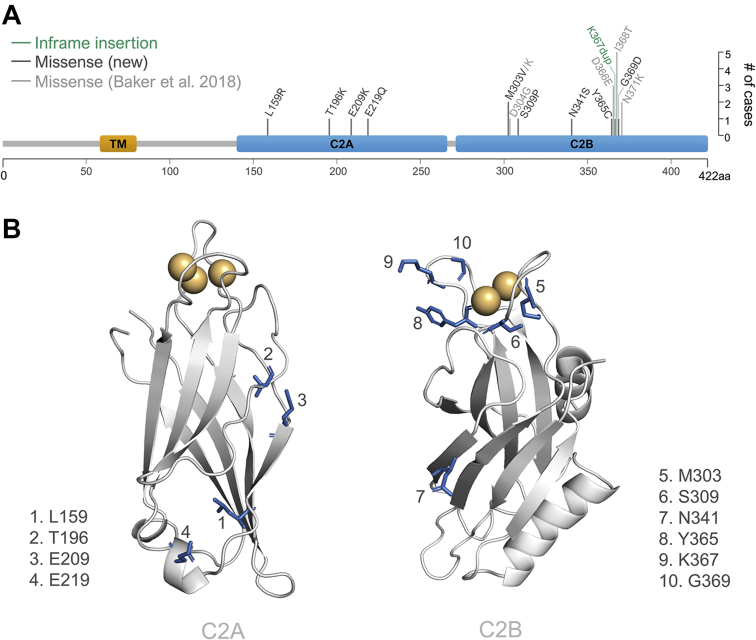
**Location of newly identified *SYT1* variants.** A. Positions and types of newly identified (dark gray) and previously described (light gray) variants are indicated on the domain structure of the *SYT1* gene. Length of the vertical line at each residue reflects the number of individual cases included in this study that harbor variants at that locus. B. Locations of the newly identified SYT1 variants are highlighted in the 3-dimensional structures of the C2A (left; Protein Data Bank [PDB]: 1BYN) and C2B (right; PDB: 1K5W) domains with residues impacted by variants shown in blue as stick representations and numerically labelled. Calcium ions (Ca^2+^) are represented as orange spheres. TM, transmembrane domain.

Overall, 15 de novo variants in *SYT1* were identified in 22 individuals (Figure 1A) (5 missense variants in 11 individuals have previously been described^8^^,^^9^). No alternative candidate variant potentially explaining neurodevelopmental presentation was identified in any case. ACMG/AMP criteria classified the variants as pathogenic (*n* = 6), likely pathogenic (*n* = 4), or uncertain (*n* = 5) (Table 1). The 11 newly reported cases comprised 1 in-frame insertion (Lys367dup) and 10 missense variants (8 at novel loci, 1 at the previously reported Met303 locus, and 1 recurrent Ile368Thr variant). All missense variants were at highly evolutionarily conserved residues from humans to invertebrates (Supplemental Table 3). Although missense variation is not constrained across *SYT1* overall (observed to expected ratio = 0.47; Genome Aggregation Database version 2.1.1,^22^ https://gnomad.broadinstitute.org/gene/syt1?dataset=gnomad_r2_1), the C2A and C2B domains lie within a region that demonstrates significant missense constraint with an observed to expected ratio of 0.24 (χ^2^ = 48.87; Exome Aggregation Consortium,^22^ https://gnomad.broadinstitute.org/gene/syt1?dataset=exac).

**Table 1:** Assessment of *SYT1* variant pathogenicity

Nucleotide and Amino Acid Change^a^	*n* in this Study	gnomAD v2.1.1	SIFT	Polyphen-2	M-CAP	Published Functional Evidence	ACMG/AMP Classification	Predicted Molecular Impact
c.476T>G p.Leu159Arg(L159R)	1	2 synonymous changes	Damaging (0)	Probably damaging (1.000)	Possibly pathogenic (0.085)	N	PM2, PM6, PP3 **VUS**	Structural perturbations in C2A
c.587C>A p.Thr196Lys(T196K)	1	2 synonymous changes (in controls)	Damaging (0)	Probably damaging (0.998)	Possibly pathogenic (0.057)	N	PM2, PM6, PP3 **VUS**	Structural perturbation in β-4 of C2A
c.625G>A p.Glu209Lys(E209K)	1	Nil	Damaging (0)	Probably damaging (0.997)	Possibly pathogenic (0.029)	N	PM2, PM6, PP3 **VUS**	Removes H-bonds with Lys197 (between β-4 and β-5)
c.655G>C p.Glu219Gln (E219Q)	1	Nil	Damaging (0)	Probably damaging (0.984)	Possibly pathogenic (0.039)	N	PM2, PP3,PM6 **VUS**	Neutralizes surface negative charge and removes salt bridge with Lys223
c.908T>A p.Met303Lys (M303K)	1	1 frameshift p.Met303TrpfsTer11 (in a neuro case)	Damaging (0)	Benign (0.329)	Possibly pathogenic (0.055)	Y^9^	PS3, PM1, PM2, PM6, PP3 **Pathogenic**	Structural perturbations in C2B
c.907A>G p.Met303Val(M303V)	1	1 frameshift p.Met303TrpfsTer11 (in a neuro case)	Tolerated (0.12)	Benign (0.322)	Possibly pathogenic (0.058)	N	PM1, PM2, PM5 (supporting), PM6 **Likely pathogenic**	Structural perturbations in Ca^2+^-binding loop 1 of C2B
c.911A>G p.Asp304Gly(D304G)	1	Nil	Damaging (0)	Possibly damaging (0.701)	Possibly pathogenic (0.229)	Y^9^^,^^10^	PS3, PM1, PM2, PM6, PP3 **Pathogenic**	Impaired Ca^2+^-binding of C2B
c.925T>C p.Ser309Pro(S309P)	1	Nil	Damaging (0)	Probably damaging (0.999)	Possibly pathogenic (0.130)	N	PM1, PM2, PM6, PP3 **Pathogenic**	Loss of H-bonds in C2B Ca^2+^-binding loop 1
c.1022A>G p.Asn341Ser (N341S)	1	Nil	Damaging (0)	Probably damaging (0.992)	Possibly pathogenic (0.239)	N	PM2, PM6, PP3 **VUS**	May perturb interaction with SNAP-25
c.1094A>G p.Tyr365Cys(Y365C)	1	Nil	Damaging (0)	Probably damaging (0.993)	Possibly pathogenic (0.144)	N	PM1, PM2, PM6, PP3 **Likely pathogenic**	Structural perturbations in Ca^2+^-binding loop 1 of C2B
c.1098C>A c.1098C>G p.Asp366Glu (D366E)	3	Nil	Damaging (0)	Probably damaging (0.997)	Possibly pathogenic (0.061)	Y^9^^,^^10^	PS3, PM1, PM2, PM6, PP3 **Pathogenic**	May alter Ca^2+^ interaction
c.1100_1102dup p.Lys367dup(K367dup)	1	Nil	-	-	-	N	PM1, PM2, PM4 (supporting), PM6 **Likely pathogenic**	Add positive charge and structural perturbations to Ca^2+^-binding loop 3 of C2B. May alter membrane interaction.
c.1103T>C p.Ile368Thr(I368T)	5	Nil	Damaging (0)	Benign (0.186)	Possibly pathogenic (0.070)	Y^10^, ^8^, ^9^	PS3, PM1, PM2, PM6, PP3 **Pathogenic**	Inhibited membrane penetration
c.1106G>A p.Gly369Asp(G369D)	1	Nil	Damaging (0)	Probably damaging (0.990)	Possibly pathogenic (0.077)	N	PM1, PM2, PM6, PP3 **Likely pathogenic**	Add negative charge to Ca^2+^-binding loop 3 of C2B. May alter membrane interaction.
c.1113C>G p.Asn371Lys(N371K)	2	49 x synonymous change (19 in controls)	Tolerated (1)	Probably damaging (0.999)	Possibly pathogenic (0.131)	Y^9^	PS3, PM1, PM2, PM6, PP3 **Pathogenic**	May perturb structure of Ca^2+^-binding loop 3 of C2B

Variant classification shown in bold.

*ACMG/AMP*, American College of Medical Genetics and Genomics/Association for Molecular Pathology; *Ca*^*2+*^, calcium ion; *gnomAD,* Genome Aggregation Database; *M-CAP*, Mendelian Clinically Applicable Pathogenicity; *N*, no; *SIFT*, Sorting Intolerant From Tolerant; *VUS*, variants of uncertain significance; *Y*, yes.

a All variants are in relation to reference sequence: NM_005639.3.

### Molecular impacts of *SYT1* variants

**Figure 2:**
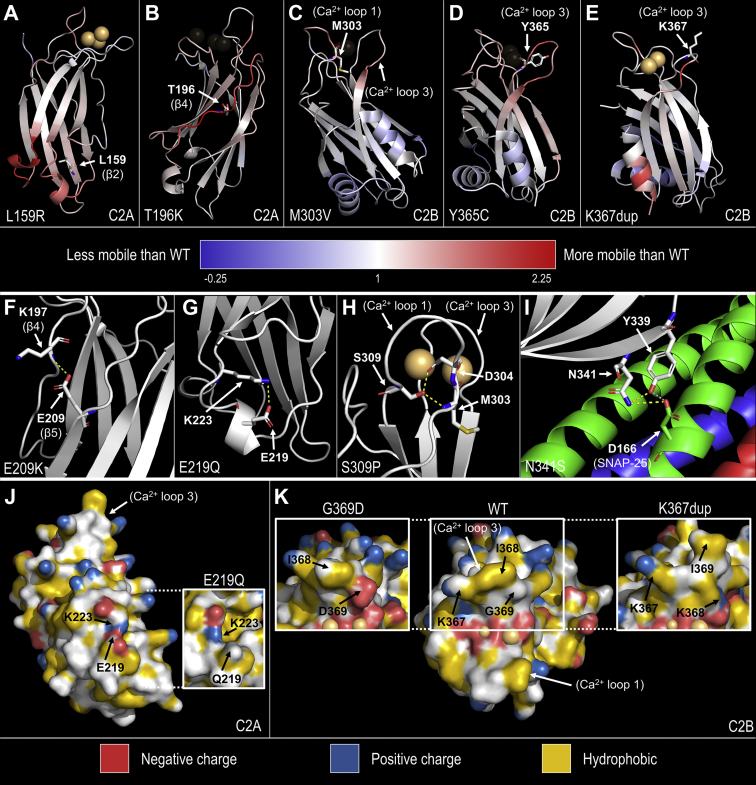
**Predicted molecular impacts of newly identified *SYT1* variants.** Variants are expected to either (A-E) alter regional mobility of the domain, (F-I) perturb intramolecular or intermolecular interactions, or (J, K) alter the surface charge of the protein. A-E. Cartoon ribbon representations of WT C2A and C2B domains where blue-white-red spectrum coloring indicates the change in mobility of each residue between WT and variant simulations. Mobility change was calculated as the average variant root-mean-square fluctuation (RMSF) (Å)/average WT RMSF (Å) for each residue, threshold at –0.25 (blue) and 2.25 (red) for illustration with 1 denoting RMSF equal to WT (white). Residues altered by *SYT1* variants are shown in stick representation and labelled. Either (A, E) Ca^2+^-bound structures or (B-D) Ca^2+^-free structures are shown to display the greatest impact on RMSF. F-H. Intramolecular interactions that were abolished in simulations of variant domains are shown as yellow dotted lines (see Supplemental Table 4 for details). F. C2A domain showing the Glu209-Thr196 hydrogen bond lost in Glu209Lys. G. C2A domain showing the Glu219-Lys223 salt-bridge lost in Glu219Gln. H. C2B domain showing Ser309-Met303 and Ser309-Asp304 hydrogen bonds lost in Ser309Pro. I. Primary interface of the SYT1–SNARE complex (Protein Data Bank: 5CCH; white: SYT1; green: SNAP-25; red: synaptobrevin-2; blue: syntaxin-1A) highlighting the intramolecular (Asn341-Tyr339 in SYT1) and intermolecular (Asn341^SYT1^-Asp166^SNAP-25^) hydrogen bonds involving Asn341 in yellow. J, K. YRB representations^23^ of Ca^2+^-bound WT and variant C2A or C2B domains (at last frame of simulation) that show surface charge and hydrophobicity (red: negative charge; blue: positive charge; yellow: hydrophobic). J. WT and Glu219Gln (inset) C2A domains. K. WT, Gly369Asp (left inset), and Lys367dup (right inset) C2B domains. Ca^2+^ ions are represented as orange spheres. Ca^2+^, calcium ion; WT, wild-type.

To further investigate the pathogenic potential of *SYT1* variants, we carried out molecular dynamics simulations of the variant C2 domains and searched for known roles of the affected residues. For all newly identified variants, the distances of Ca^2+^ ions from a reference amino acid in the Ca^2+^-binding pocket were similar to the WT simulations (Supplemental Figure 1), indicating that disturbed Ca^2+^ retention is unlikely to be a major pathogenic mechanism. In contrast, simulations of the previously reported Asp304Gly variant recapitulated defects in the retention of Ca^2+^ (Supplemental Figure 1).^9^ Although no variant caused major structural changes to the C2 domain (Supplemental Figure 2), many variants altered the mobility of discrete regions of the domains (Figure 2, Supplemental Figures 3 and 4) or would be expected to impact intramolecular or intermolecular interactions (Figure 2, Supplemental Table 4). These results suggest that these newly identified variants may impact the structure or function of SYT1 through diverse molecular mechanisms. Literature-informed predictions of molecular impacts and molecular dynamic simulation results are described later for each newly reported variant.

All 4 C2A substitutions occurred in regions of undetermined function. Leu159, lying in β-2, faces the hydrophobic interior of the domain. The side chain of the introduced charged arginine (Leu159Arg) inserts between the β-sheets and disrupts the stability of the region with elevated RMSF in multiple regions, particularly across the “bottom” (non-Ca^2+^-binding) loops of the C2A domain (Figure 2A, Supplemental Figure 3). Two substitutions, Thr196Lys and Glu209Lys, are in close proximity to each other in opposing β-strands (β-4 and β-5, respectively) on the edge of the C2A β-sandwich (Figure 1B). Thr196 is structurally important with its side chain buried between β-3 and β-4, inducing a distortion in the β-sheet structure.^24^ Thr196Lys exhibits substantially increased RMSF over residues Lys190 to Lys201 (Figure 2B, Supplemental Figure 3), indicating localized instability possibly arising from impaired anchoring of β-4 to the rest of the β-sheet. Glu209 forms hydrogen bonds with Lys197 in β-4 of the opposing β-sheet that are removed by lysine substitution (Figure 2F, Supplemental Table 4). However, there is no observable alteration in the RMSF of the Glu209Lys variant (Supplemental Figure 3). Glu219 is situated in a short alpha helix distal to the Ca^2+^-binding loops; its side chain points away from the protein and could possibly participate in intermolecular interactions. Glu219Gln neutralizes the negative charge at that site and removes a salt bridge between Glu219 and Lys223 (Figure 2G and J, Supplemental Table 4) but does not result in an appreciable effect on the regional RMSF (Supplemental Figure 3).

Similar to previously reported cases,^9^ all newly identified C2B missense variants map to the region surrounding the Ca^2+^-binding pocket (Met303Val and Ser309Pro in loop 1; Tyr365Cys, Lys367dup, and Gly369Asp in loop 3) with a notable exception, Asn341Ser, which is located in a β-strand (Figure 1B).

Met303 anchors Ca^2+^-binding loop 1, and replacement with valine is predicted to render the loop more flexible, with increased RMSF across residues 301 to 306 in Ca^2+^-free Met303Val simulations (Figure 2C, Supplemental Figure 4). Ser309Pro would abolish transient hydrogen bonds between Ser309 and Met303 and between Ser309 and Asp304 that are normally present in the Ca^2+^-bound WT C2B domain (Figure 2H, Supplemental Table 4), but no change to the RMSF of Ca^2+^-binding loop 1 was observed in Ser309Pro simulations (Supplemental Figure 4). Notably, a pathogenic variant has been found at the residue corresponding to Ser309 in the homologous *SYT2*.^25^ In the Ca^2+^-bound simulations, both Met303Val and Ser309Pro increased the mobility of the distal arginine apex (Arg399, Arg400) at the opposite end of the C2B domain (Supplemental Figure 4).

No structural alterations were detected in simulations of the Asn341Ser variant (Supplemental Figure 4), but importantly, Asn341Ser may perturb the interaction between SYT1 and the SNARE complex. Asn341 faces outward on β-5 and is proximate to the primary binding interface between SYT1 C2B and SNAP-25.^26^ Crystal structures of the SYT1–SNARE complex (Protein Data Bank: 5CCH, 5CCG, and 5KJ7)^26^ show that Asn341 may interact directly with Asp166 of SNAP-25 or form a hydrogen bond with the neighboring Tyr339 in SYT1, which binds to Asp166 of SNAP-25 (Figure 2I). Interestingly, 2 variants at Asp166 have been identified in individuals with *SNAP25* developmental and epileptic encephalopathy.^27^

Tyr365 stabilizes Ca^2+^-binding loop 3, and the mobility of this loop is increased in Tyr365Cys variant simulations (Figure 2D, Supplemental Figure 4). Furthermore, Asp366 flips out of the Ca^2+^-binding pocket, and Lys367 twists to impinge on the Ca^2+^-binding pocket in Ca^2+^-free Tyr365Cys simulations. The positive charge at Lys367 is important for phospholipid binding.^28^, ^29^, ^30^ The Lys367dup variant introduces an additional positively charged lysine to Ca^2+^-binding loop 3 (Figure 2K), which could potentially increase attraction between the tip of loop 3 and anionic phospholipids. In addition, simulations showed increased flexibility in loop 3 (Figure 2E, Supplemental Figure 4) and transient interactions between the inserted lysine and Ca^2+^-coordinating residues Asp366, Asp373, Asp310, and Asp304. Gly369Asp introduces an additional negative charge to the Ca^2+^-binding pocket (Figure 2K), more specifically, to the membrane-penetrating tip of loop 3, which could be expected to repel the anionic plasma membrane.

### Clinical histories

The most common features within the SYT1 group were developmental delay, abnormal eye physiology, and an abnormal EEG (cohort summary in Table 2; individual data in Supplemental Table 2). There is a wide range of severity of neurodevelopmental impairments––approximately one-third of cases presented with mild or moderate delay to motor and communication milestones, whereas almost all previously reported cases were severely delayed.^8^^,^^9^ Other common features were sleep disorders, feeding difficulties, gastrointestinal reflux, and finger chewing or other self-injury, each affecting around two-thirds of cases.

**Table 2:** Clinical phenotypes summary

*Clinical feature* HPO Term Identifier^a^	Data Available (*n*)	Frequency of Feature (*n*)	Frequency of Feature (%)	Subtype (*n*)
Delayed speech and language development HP:0000750	22	21	95	Mild = using words and phrases (5); moderate = using single words only (2); severe = not using any words (6); unable to classify as under age 5 years or insufficient information (8)
EEG abnormality HP:0002353	18	17	94	Ictal features (8); intermittent low frequency oscillations (8); abnormal background activity unspecified or generalized slowing (6)
Abnormal eye physiology HP:0012373	22	20	91	Strabismus/esotropia (12); nystagmus (6); hypermetropia (3); visual impairment unspecified (3)
Neonatal hypotonia HP:0001319	22	19	86	-
Motor delay HP:0001270	22	18	81	Mild = walked by 3 years (4); moderate = walked by 5 years (3); severe = walked after 5 years or nonambulatory over the age of 5 (6); unable to classify because nonambulatory under age 5 years (5)
Abnormality of movement HP:0100022	21	14	66	Dystonia (7), chorea (7), dyskinesia (1), ataxia (5), myoclonus (3), tremor (2), stereotypies (6)
Sleep disturbance HP:0002360	19	12	63	Commonly hypersomnia during infancy then difficulties initiating and maintaining sleep
Abdominal symptom HP:0011458	22	13	59	Feeding difficulties (8), gastroesophageal reflux (6), drooling (2), constipation (3), chronic diarrhea (1), urinary retention (2), pancreatitis with pseudocysts (1)
Self-injurious behavior HP:0100716	22	13	59	Finger biting or chewing (9), head banging (3), skin picking (1), other or unspecified (1)
Abnormality of the musculoskeletal system HP:0033127	22	8	36	Torticollis (1), joint hypermobility (2), talipes (2), pes planovalgus (1), progressive contractures (1), scoliosis (1)
MRI abnormality HP:0012639	15	5	33	Mild diffuse progressive volume loss (1), delayed myelination (2), mild periventricular white matter nodular abnormality (2)
Abnormality of the respiratory system HP:0002086	22	6	27	Sleep apnea (4), laryngomalacia (1), hyperventilation with cyanosis (1), autonomic dysfunction with hypotension (1)
Phenotypic abnormality (other) HP:0000118	22	5	22	Undescended testicle (1), atrial septal defect (1), dermoid cyst (2), unilateral syndactyly (1)
Seizure HP:0001250	22	4	18	Absence seizures (3), tonic-clonic seizures (1), infantile spasms (1)
Abnormality of prenatal development or birth HP:0001197	22	3	13	Mild prematurity (1), neonatal resuscitation (1), meconium aspiration (1)

*EEG,* electroencephalogram.

a Human Phenotype Ontology^31^ (https://hpo.jax.org/).

Movement disorder was a feature in two-thirds of the cohort. The types and severities of involuntary movements were variable and included ataxia, tremor, and myoclonus as well as dystonia, chorea, and complex hyperkinetic movement disorders in more severely affected cases.

Contrasting our previous case series with no cases of epilepsy,^9^ 4 individuals in the current group had received an epilepsy diagnosis. The reported seizure phenotypes included absences (*n* = 3), tonic-clonic seizures (*n* = 1), and infantile spasms (*n* = 1).

EEG abnormalities remain very common across the cohort, encompassing low-frequency background oscillations as previously reported but also ictal features in individuals with and without overt seizures.

Although most individuals within the group were young children, it is possible to make some preliminary comments about the longer-term trajectory of the condition based on 9 individuals who are currently older than 10 years. We note the potential for long-term positive progress in motor development with 2 individuals learning to walk after the age of 5 years. However, 2 individuals have developed movement disorder symptoms during late childhood, accompanied by a relative decline in adaptive skills. We also observed that social and emotional difficulties may emerge with time, with some older children and adolescents experiencing obsessions, anxieties, and mood disturbance.

### Behavioral phenotyping and comparison with an ID control group

**Figure 3:**
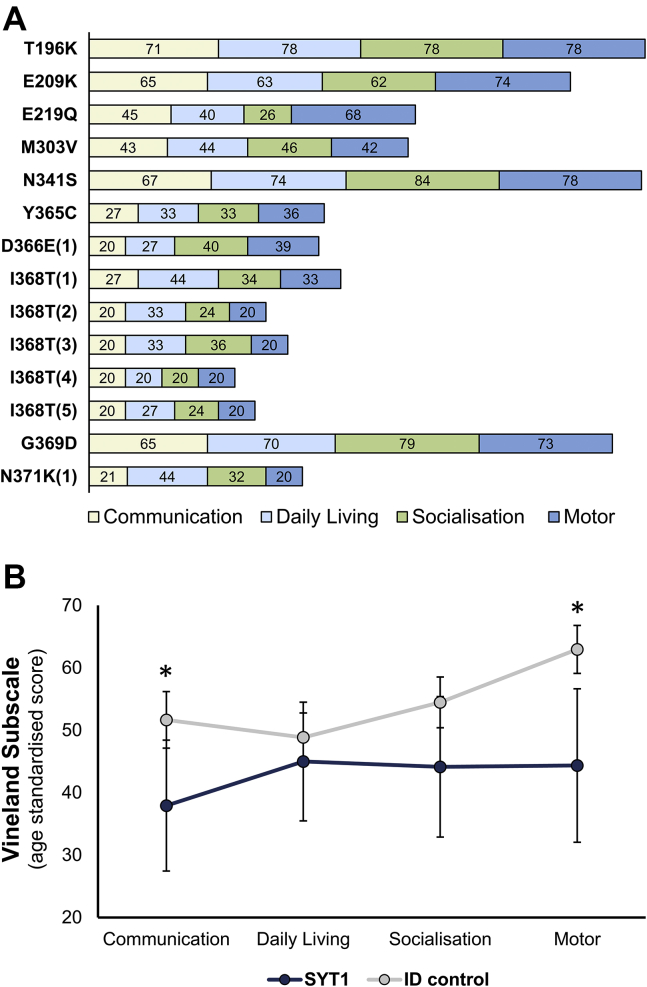
**Adaptive behavior profiles of SYT1 and comparison groups.** A. Standardized scores for Vineland Adaptive Behavior Scales subdomains are shown for each SYT1 individual assessed. Case numbers corresponding to individuals listed in Supplemental Table 2 are indicated in parentheses for recurrent variants. B. Comparisons of group averages of Vineland subdomains for SYT1 (*n* = 14) and ID control (*n* = 51) groups (uncorrected for Vineland form). Error bars represent 95% CIs. General linear model analysis revealed significant differences between groups in the communication and motor subdomains (∗*P* < .05). ID, intellectual disability.

The SYT1 and ID comparison groups did not significantly differ in the range and distributions of age, sex, and global adaptive function (Supplemental Table 5). Vineland Adaptive Behavior Composite scores within the SYT1 group ranged from 20 to 74. Four participants scored in the borderline or mild ID range, 3 in the moderate ID range, and 7 in the severe or profound ID range (see Figure 3A for breakdown of subscale scores for each individual). Inspecting Vineland subscale scores within the SYT1 group, we observed that, on average, communication ability was more severely impaired than motor abilities, socialization, and daily living skills. In contrast, motor ability was a relative strength within the ID comparison group. To establish whether these differences reflect a consistent and discriminating profile of adaptive functions within the SYT1 group, we carried out general linear model analysis (within-subjects factor: Vineland subscale score; between-subjects factors: group). This highlighted significant interaction between group and subscale (*F* = 4.54, *df* = 2.33 Greenhouse Geisser corrected, *P* = .01; reduced to *P* = .09 after co-varying for Vineland edition). Post hoc nonparametric analyses indicated significantly lower scores for the SYT1 group in motor ability (*P* = .036) and communication (*P* = .019) but not for socialization (*P* = .07) or daily living skills (*P =* .28) (Figure 3B).

We next explored DBC-P and Social Responsiveness Scale 2 scores, assessing emotional or behavioral problems and autism-related social impairment, respectively. No significant differences emerged between the SYT1 and ID comparison groups for total scores on either measure, and general linear model analyses identified no significant group × subscale interaction for either measure (Supplemental Figures 5 and 6).

In our previous description of *SYT1*-associated phenotypes,^4^ based on clinical reports only, we noted that a high proportion of diagnosed individuals displayed motor stereotypies, unpredictable mood switches, and episodes of agitation. To explore whether these features were increased in the expanded *SYT1*-diagnosed population, beyond expectation for ID, 5 relevant items reflecting these symptoms were selected from the DBC-P (6: bangs head; 10: chews or mouths body parts; 33: hits or bites self; 47: mood changes rapidly for no reason; and 60: has repeated movements of hands, body, or head). These items appear across 2 different DBC-P subscales (disruptive/antisocial, self-absorbed). The raw scores (0-2) for each item were summed for each participant. Univariate analysis, co-varying for age, identified significantly higher scores for these selected DBC items within the SYT1 group (SYT1 group: M = 5.57; SD = 2.95; range = 0-9; ID comparison group: M = 3.67; SD = 2.98; range = 0-10; *F* = 5.95, *df* = 1, *P* = .02). The age-adjusted summed scores showed a strong negative correlation with the Vineland Adaptive Behavior Composite scores within the SYT1 group (Spearman’s rho = –0.73; *P* = .003), but not the comparison group (Spearman’s rho,= –0.18; *P* = .26; Fisher’s test^32^ *z* = –2.19; *P* = .01) (Supplemental Figure 7). In summary, self-injury, mood instability, and repetitive movements are elevated within the SYT1 group with a strong relationship between these features and global adaptive impairment.

## Discussion

This study builds on the previous identification and characterization of *SYT1*-associated neurodevelopmental disorder to broaden the range of potentially pathogenic variants for clinical laboratory reporting. We detailed the expansion of the genotypic and phenotypic spectrum of this syndrome with the objectives of increasing the diagnostic efficiency of this ultrarare disorder and improving the prognosis estimation, patient management, and information available for families and clinicians after diagnosis.

### Broadening the genetic landscape

We describe 4 novel variants in the SYT1 C2A domain as well as additional C2B loci. Although the 4 C2A variants and 1 C2B variant remain of uncertain significance according to ACMG/AMP criteria, and functional evidence is required to substantiate pathogenicity, molecular dynamics simulations and existing literature support potential functional impacts. Novel variants may exert dominant-negative effects, as seen for previously identified variants,^10^, ^8^, ^9^ but we cannot preclude haploinsufficiency as a possible pathogenic mechanism. Ca^2+^-binding loops 1 and 3 of the C2B domain seem to be highly sensitive to variation as pathogenic missense variants cluster in these regions in both *SYT1* and the homologous *SYT2*,^25^^,^^33^^,^^34^ and no missense *SYT1* variants in these loops are recorded in the Genome Aggregation Database version 2.1.1.^22^ Therefore, any de novo missense variant in the C2B Ca^2+^-binding loops should be investigated for possible pathogenicity, and variants in other highly conserved residues of the SYT1 C2 domains should also be considered.

### Broadening the phenotypic spectrum

The major clinical features associated with *SYT1* variants in this larger cohort are broadly in keeping with those reported in the previous case series.^9^ *SYT1*-associated neurodevelopmental disorder presents with individually nonspecific features, but may be suspected when neonatal hypotonia, developmental delay, abnormal eye physiology, movement disorders, and EEG abnormalities are present in any combination. Although some of the newly identified cases present with profound developmental delay and involuntary movement disorders, in line with the presentation of the initial cases, others show milder neurodevelopmental difficulties, thereby widening the range of clinical severity compatible with this diagnosis. For these less severe cases, it is more difficult to distinguish a discriminating phenotypic signature to aid in variant interpretation and diagnostic confirmation. Additional cases are required to clarify the association between *SYT1* and epilepsy risk. Given the young age of some individuals within the cohort and uncertainty of the developmental trajectory of this disorder, a cautious prognosis is warranted. We note that among some older diagnosed cases, later-onset movement disorder and decline in adaptive functioning and emotional wellbeing have been observed. Longitudinal sampling of individuals with *SYT1* variants will facilitate mapping of the developmental progression of this disorder and improve prognosis estimations.

Questionnaire data revealed that all domains of adaptive function are impaired within the SYT1 cohort, and highlighted a disproportionate impact on motor and communication function when compared with a comparison ID group (however, note that data could not be collected for 7 SYT1 cases due to language barriers or young age). Moreover, features of rapid mood change and motor stereotypies (specifically hand biting) are prominent, which is in line with previous observations.^9^ Improved understanding of the cell type–specific dependence on SYT1 for efficient neurotransmitter release will aid in symptom explanation at the circuit and large-scale network levels. Future investigations will involve additional parent/carer-report questionnaires to further probe the neurological and behavioral domains impacted by *SYT1* variants, such as visual behavior, movement disorder symptoms, repetitive behaviors, and hyperactivity.

With expansion of the cohort, *SYT1*-associated neurodevelopmental disorder continues to demonstrate intersecting clinical features that are common among synaptic vesicle cycling disorders.^3^ However, seizures are a significant feature of disorders associated with pathogenic variants in other core synaptic vesicle fusion machinery including *VAMP2*, *STX1B*, *SNAP25*, *STXBP1*, *CPLX,1* and other accessory proteins.^35^^,^^36^ In notable contrast, although seizures have been reported for 4 SYT1 cases, epilepsy is evidently not a prevalent or prominent feature of this disorder. While EEG abnormalities were frequent within the SYT1 group, the electrophysiological features observed were variable and there were inconsistencies in the reporting methods, provision of recordings for review, patient age, and conditions of recordings. Standardized and systematic EEG data collection and analysis are needed to confirm common electrophysiological characteristics and inform understanding of the neurophysiological origins of symptoms. Furthermore, systematic behavioral characterization of other disorders of fusion, and synaptic vesicle cycling disorders more broadly, will allow a more detailed comparison of these mechanistically-related syndromes and interrogation of distinctive characteristics of this group of disorders.

### Diversity of molecular mechanisms

Molecular dynamics simulations and SYT1 structure-function relationships sourced from the literature were used to carefully consider variants in the context of the 3-dimensional protein structure. The newly identified variants are predicted to have nuanced effects on the local stability of discrete protein regions (including Ca^2+^-binding loops or β-sheets), penetration of the phospholipid membrane, or interaction with SNARE proteins, rather than cause gross destabilization of the C2 domain or substantial impairment of Ca^2+^ binding. Such perturbations could conceivably disrupt SYT1 function and synaptic transmission. Some disorder-associated variants, particularly those within the C2A domain, provide the first indication of the importance of previously unrecognized residues and regions of SYT1. It should be noted that although molecular dynamics simulations provide atomic-level predictions of variant impact on protein structure and Ca^2+^ retention, these simulations are limited in length and unable to reveal impacts on protein-protein or protein-lipid interactions or consequences on neurotransmission. Functional studies at the molecular, cellular, and circuit levels will provide further evidence for pathogenicity and insight into the specific mechanisms underlying neurodevelopmental impairments.

### Genotype-phenotype links

We inspected the current data sets for any evidence that the specific *SYT1* variant may contribute to clinical features and severity. Questionnaire data were available for 5 cases with the recurrent Ile368Thr variant, which revealed that the neurological and behavioral phenotype was highly consistent between these individuals (Figure 3, Supplemental Table 2). Although questionnaire data were not available for other recurrent variants, clinical reports of recurrent locus Met303 and variants Asp366Glu and Asn371Lys show similar consistency in phenotype severity (Supplemental Table 2). This raises the possibility of a relationship between the diversity of molecular mechanisms and phenotypic variation. No obvious patterns emerged between the clinical phenotype and either the nature of the amino acid substitution or the conservation of the affected residue across synaptotagmin isoforms.

We subsequently asked whether there are consistent differences between C2A and C2B domain variants in the global severity of impairments. While acknowledging that cases of C2A variants are limited and remain of uncertain significance, it is notable that all 4 individuals with C2A variants present with mild or moderate adaptive impairments and the absence of early-onset movement disorder. Correspondingly, the variants linked to the most severe clinical phenotypes are all situated in the C2B domain (although phenotypes associated with C2B variants are not universally severe). The prospect of disparity in clinical severity between the C2A and C2B domains is congruent with the theory that each C2 domain may play different roles in the functions of SYT1.^37^^,^^38^ In addition, the location of variants within each domain differs, with most of the C2B variants, but no C2A variants, located in the Ca^2+^-binding loops. Identification of additional variants and detailed phenotyping of recurrent variants will assist in clarifying genotype-phenotype relationships. Functional studies are required to confirm the pathogenicity of C2A variants and identify mechanisms contributing to the variation in clinical severity. The impacts of variants on all functions of SYT1, not only on evoked exocytosis but also suppression of spontaneous and asynchronous release and modulation of endocytosis,^39^^,^^40^ need to be investigated to fully appreciate the similarities and differences in the molecular and cellular impacts. Integration of genetic, cellular, neural systems and cognitive investigations will enable a thorough understanding of *SYT1*-associated neurodevelopmental disorder with the prospect of precision medicine targeting each individual’s symptoms and underlying mechanisms.

## Data Availability

Reporting of *SYT1* variants in open access repositories is listed in Supplemental Table 2. Molecular dynamics simulation data not included in the Supplemental Material are available upon request from the corresponding author.

## Conflict of Interest

The authors declare that there are no commercial associations that might pose or create the appearance of a conflict of interest with the information presented in this manuscript.

## Supplementary Material

Supplemental Table 2

Supplemental Table 2 References
