# Supplementary material for: Expanding the genotype and phenotype spectrum of SYT1-associated neurodevelopmental disorder
Source: Genet Med. 2022 Apr;24(4):880–93. doi: 10.1016/j.gim.2021.12.002 (PMC8986325; doi:10.1016/j.gim.2021.12.002)
Supplement: Supplemental Material [file mmc1.pdf]

Supplementary Material

Expanding the genotype and phenotype spectrum of *SYT1*-associated neurodevelopmental disorder

Holly Melland[1,2], Fabian Bumbak[3], Anna Kolesnik-Taylor[4], Elise Ng-Cordell[4], Abinayah John[4], Panayiotis Constantinou[5], Shelagh Joss[5], Martin Larsen[6], Christina Fagerberg[6], Lone Walentin Laulund[7], Jenny Thies[8], Frances Emslie[9], Marjolein Willemsen[10], Tjitske Kleefstra[10,11], Rolf Pfundt[10], Rebekah Barrick[12], Richard Chang[12], Lucy Loong[13], Majid Alfadhel[14,15,16], Jasper van der Smagt[17], Mathilde Nizon[18], Manju Kurian[19], Daniel J Scott[1], Joshua J Ziarek[3], Sarah Gordon[1,2], Kate Baker[4,20]✉.

[1]The Florey Institute of Neuroscience and Mental Health, University of Melbourne, Parkville, VIC, Australia. [2]Melbourne Dementia Research Centre, The Florey Institute of Neuroscience and Mental Health, University of Melbourne, Parkville, VIC, Australia. [3]Department of Molecular and Cellular Biochemistry, Indiana University, Bloomington, IN, USA [4]MRC Cognition and Brain Sciences Unit, University of Cambridge, Cambridge, UK. [5] Department of Clinical Genetics, Queen Elizabeth University Hospital, Glasgow, UK. [6] Department of Clinical Genetics, Odense University Hospital, Odense, Denmark. [7] H C Andersen Children's Hospital, Odense University Hospital, Denmark. [8] Department of Pediatrics, Division of Genetic Medicine, Seattle Children's Hospital, Seattle, WA, USA. [9] South West Thames Regional Genetics Service at St George's, University of London, London, UK. [10] Radboud University Medical Center, Nijmegen, NL. [11] Vincent van Gogh Centre for Neuropsychiatry, Venray, NL. [12] Children's Hospital of Orange County, Orange, CA, USA. [13] Oxford Centre for Genomic Medicine, Oxford University Hospitals NHS Foundation Trust, Oxford, UK. [14] Genetics and Precision Medicine department, King Abdullah Specialized Children Hospital, King Abdulaziz Medical City, Ministry of National Guard Health Affairs, Riyadh, Saudi Arabia. [15] Medical Genomics Research Department, King Abdullah International Medical Research Center, Ministry of National Guard Health Affairs, Riyadh, Saudi Arabia. [16] College of Medicine, King Saud bin Abdulaziz University for Health Sciences, King Abdulaziz Medical City, Ministry of National Guard Health Affairs, Riyadh, Saudi Arabia. [17] Utrecht University Medical Centre, Utrecht, NL. [18] CHU Nantes, Service de Génétique Médicale, INSERM, Université de Nantes, Nantes, France. [19] Developmental Neurosciences Programme, UCL Institute of Child Health, London, UK. [20] Department of Medical Genetics, University of Cambridge, Cambridge, UK. ✉email: Kate.baker@mrc-cbu.cam.ac.uk

Supplementary Methods

Evaluation of Variants

gnomAD v2.1.1 (The Genome Aggregation Database) (https://gnomad.broadinstitute.org/) was searched to identify allele frequency (AF) of reported variants in control populations. To identify the evolutionary conservation of the amino acids in the protein sequence across 14 species from Human to *Caenorhabditis elegans*, we employed **COBALT** (Constraint-based Multiple Alignment Tool) (https://www.ncbi.nlm.nih.gov/tools/cobalt/cobalt.cgi). We deployed three *in-silico* predictive programmes, namely **SIFT version 5.2.2** (Sorting Intolerant From Tolerant) (http://sift.jcvi.org) to predict the effect of amino acid substitution on protein function, **PolyPhen-2** (Protein Analysis Through Evolutionary Relationship) (http://genetics.bwh.harvard.edu/pph2/) to predict deleteriousness of single nucleotide variants and insertions/deletions variants, **M-CAP** (Mendelian Clinically Applicable Pathogenicity) (http://bejerano.stanford.edu/mcap/) to combine previous pathogenicity scores (SIFT, PolyPhen) to increase the overall sensitivity. Evidence for pathogenicity and classification were carried out independently by two authors. Phenotypic similarity (supporting criterion PP4 in Richards et al. 2015[1]) was not applied, to avoid bias of our analysis of phenotypic spectrum.

Molecular Dynamics Simulations

MD simulations were carried out based on NMR structures of the synaptotagmin-1 (SYT1) C2A (PDB: 1BYN[2]) and C2B (PDB: 1K5W[3]) domains, or variants thereof, either in the presence or absence of bound Ca^{2+} atoms. Single variant homology models of SYT1 C2A comprising variants Leu158Arg, Thr195Lys, Glu208Lys and Glu218Gln, and SYT1 C2B comprising variants Met302Val, Asp303Gly, Asn340Ser, Tyr364Cys and Gly368Asp were generated using Swiss-PdbViewer v4.1.0, while the homology model containing the Lys366 duplication (K366dup) was generated using SWISS-MODEL[4] (note that numbering used throughout this paper follows human sequence for simplicity (i.e. Leu159Arg, Thr196Lys, Glu209Lys and Glu219Gln, Met303Val, Asp304Gly, Asn341Ser, Tyr365Cys and Gly369Asp). The SYT1 C2A models were C- and N-terminally capped with Ace and NMe groups, respectively, to eliminate aberrant interactions with Ca3. Ca^{2+} atoms were removed from homology models prior to equilibration for simulations without Ca^{2+}. All MD simulations were performed with the Gromacs molecular dynamics simulation package running version 2019.4 or 2020.3[5] using the Amber ff99SB-ILDN forcefield[6] under periodic boundary conditions and in a rhombic dodecahedron unit cell. Each system was solvated with simple point charge (SPC) water molecules[7] and Na^+ and Cl^- ions were added to neutralize the total charge of the systems at concentrations of 150 mM. The neighbour list, Coulomb and van der Waals interaction cut-offs were set to 1 nm and the particle mesh Ewald (PME) algorithm[8] was utilised for long-range electrostatic interactions. All systems were first subjected to a 2000 step steepest descent energy minimization, alternatively completing when the maximum force on any atom has reached 1000 kJ mol^{-1} nm^{-1}. A 1 ns NVT (constant Number of particles, Volume and Temperature) was then performed, heating the systems to 310 K using the V-rescale thermostat[9] with position restraints on. This was followed by a 1 ns NPT (constant Number of particles, Pressure and Temperature) equilibration run performed with position restraints on using the V-rescale thermostat[9] and the Berendsen barostat[10]. A third equilibration phase using randomised initial velocities, the V-rescale thermostat[11] (310 K) and the Parrinello-Rahman barostat[12] was performed for 10 ns using a 2 fs time step. Four individual production runs of ~400ns were carried out for each system under the same conditions as the final equilibration phase but with position restraints switched off. We expected to observe nuances across four simulations that may not be observable in a single trajectory and, importantly, results for WT and Asp304Gly structures obtained with these four shorter simulations are in good agreement with previous results for single ~1200ns trajectories based on the same starting models[13]. The MD trajectories were simplified by extracting every nanosecond and by removing all water molecules using the GROMACS trjconv utility. All simulations were carried out on the 'Big Red 3' system provided by the Indiana University Pervasive Technology Institute, Bloomington IN. Data analysis was carried out in VMD (v1.9.3) using the RMSF, RMSD and hydrogen bond (cut-off set to 3.5Å and 20˚) plugins and Graphpad Prism (v9.0.0), and images of molecular snapshots were generated with PyMOL (v2.4.0). To measure Ca^{2+} retention, the distance between each Ca^{2+} atom and the C^{γ} of Asp231 of C2A or Asp364 of C2B was measured for all trajectories and plotted as a function of simulation time. Asp231 and Asp364 were used as reference amino acids as they are directly involved in electrostatic interactions with Ca1 and Ca2 in each C2 domain and are the most stable among Ca^{2+}-interacting residues. While Ca1 and Ca2 of both C2 domains remained stably bound in WT simulations (Figure S1), Ca3 occupancy of the C2A Ca^{2+}-binding pocket was remarkably unstable with Ca3 dissociating from the WT domain at early timepoints in all four trajectories (Figure S8). Ca3 also dissociated from each variant C2A domain (Leu159Arg, Thr196Lys, Glu209Lys, Glu219Gln) in at least one trajectory (Figure S8). Early Ca3 dissociation is reasonable given the low affinity of Ca3 (K_d of >>1mM)[14,15], and it is unlikely that any of the altered residues make a significant contribution towards stabilising Ca3 as a correlation with RMSF values of residues involved in Ca3 binding could not be established.

Supplementary Data

Supplementary Table 1. Genetic diagnosis summary for the ID comparison group.

Gene	n	%
ARID1B	11	21.6
CASK	2	3.9
CTNNB1	1	2.0
DDX3X	15	29.4
DLG3	2	3.9
DYRK1A	3	5.9
EHMT1	5	9.8
GRIN2A	1	2.0
KAT6B	1	2.0
PAK3	1	2.0
SETD5	6	11.8
SHANK3	2	3.9
SMARCA2	1	2.0
	=51	

Supplementary Table 2. Clinical descriptions of individual SYT1 cases. – *Available for download separately as Excel Spreadsheet.*

Supplementary Table 3. Evolutionary conservation at sites of novel *SYT1* variants. Sequence alignment performed with COBALT (https://www.ncbi.nlm.nih.gov/tools/cobalt/cobalt.cgi). Patient variants are shown in red, while blue highlights amino acids that are not identical to the human sequence.

	L159	T196	E209	E219	M303	D304	S309	N341	Y365	D366	I368	G369	N371
Variant	R	K	K	Q	K/V	G	P	S	C	E	T	D	K
H. sapiens	L	T	E	E	M	D	S	N	Y	D	I	G	N
P. troglodytes	L	T	E	E	M	D	S	N	Y	D	I	G	N
M. mulatta	L	T	E	E	M	D	S	N	Y	D	I	G	N
M. musculus	L	T	E	E	M	D	S	N	Y	D	I	G	N
R. norvegicus	L	T	E	E	M	D	S	N	Y	D	I	G	N
C. familiaris	L	T	E	E	M	D	S	N	Y	D	I	G	N
F. cattus	L	T	E	E	M	D	S	N	Y	D	I	G	N
B. taurus	L	T	E	E	M	D	S	N	Y	D	I	G	N
S. scrofa	L	T	E	E	M	D	S	N	Y	D	I	G	N
G. gallus	L	T	E	E	M	D	S	N	Y	D	I	G	N
X. tropicalis	L	T	E	E	M	D	S	N	Y	D	I	G	N
D. rerio	L	T	E	E	M	D	S	N	Y	D	I	G	N
D. melanogaster	L	T	E	D	M	D	S	N	Y	D	I	G	S
C. elegans	L	T	E	E	M	D	S	N	Y	D	L	G	N

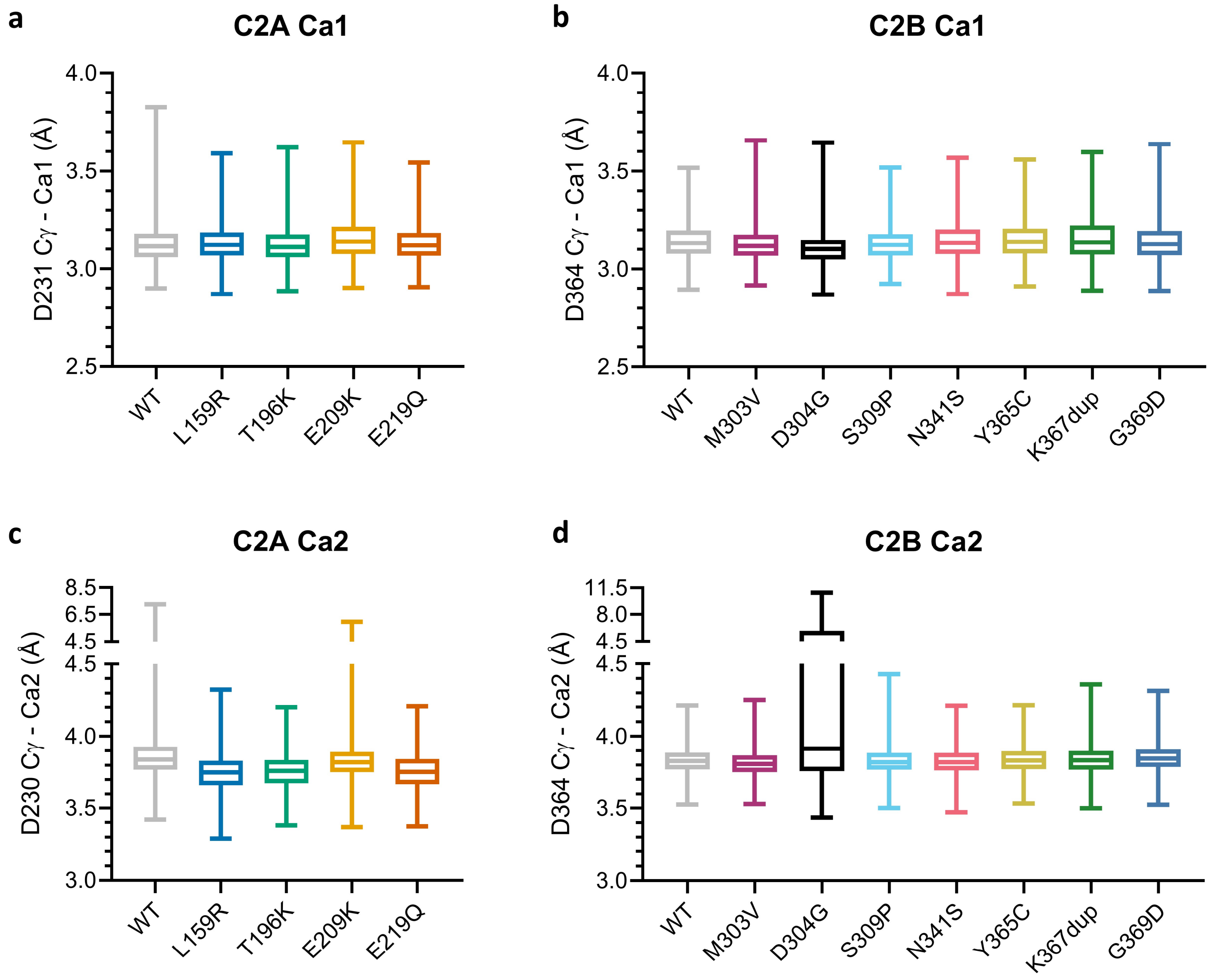

Supplementary Figure 1. Retention of Ca^{2+} ions in binding pocket of SYT1 variants. Ca^{2+}-bound models of WT and variant SYT1 C2A and C2B domains each underwent four ~400ns molecular dynamics simulations. Retention of bound Ca^{2+} ions was assessed by measuring the distance between each Ca^{2+} atom and the Cγ of Arg231 of C2A (**a**,**c**) or Arg364 of C2B (**b**,**d**) in each simulation frame. Box and whisker plots show median, first and third quartiles, and minimum and maximum distances of all four trajectories per variant. No variant substantially affected Ca^{2+} retention except for the control variant Asp304Gly, as previously shown by Baker et al. (2018)[13].

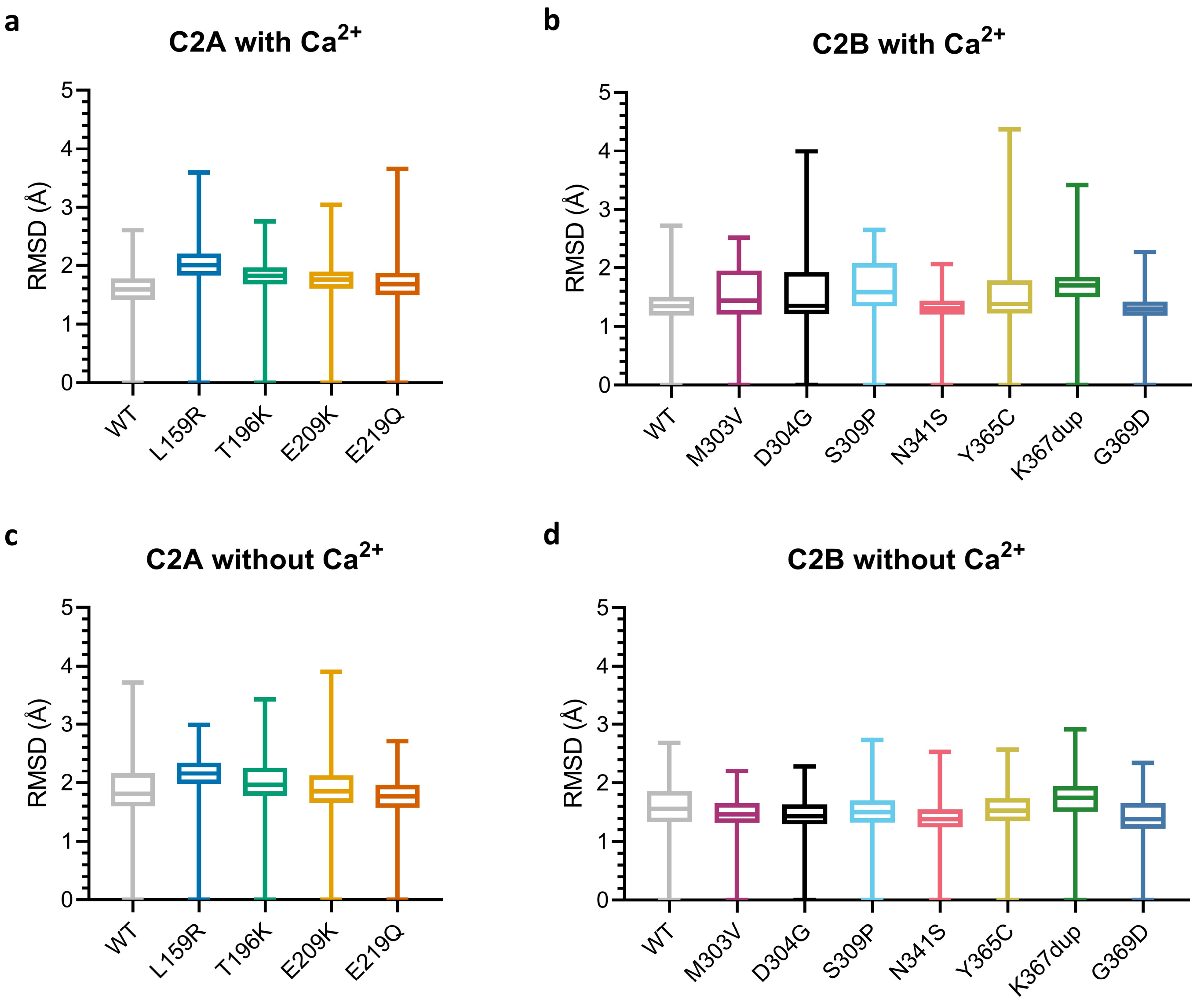

Supplementary Figure 2. RMSD of C2A and C2B variants. Models of WT and variant SYT1 C2A (**a,c**) and C2B (**b,d**) domains, with Ca^{2+} ions either present (**a,b**) or removed (**c,d**), each underwent four ~400ns molecular dynamics simulations. RMSD (root-mean-square deviation) of the backbone atoms of each domain variant, compared to the starting structure, was measured over the course of each simulation. Box and whisker plots show median, first and third quartiles, and minimum and maximum RMSD values of all four trajectories per variant.

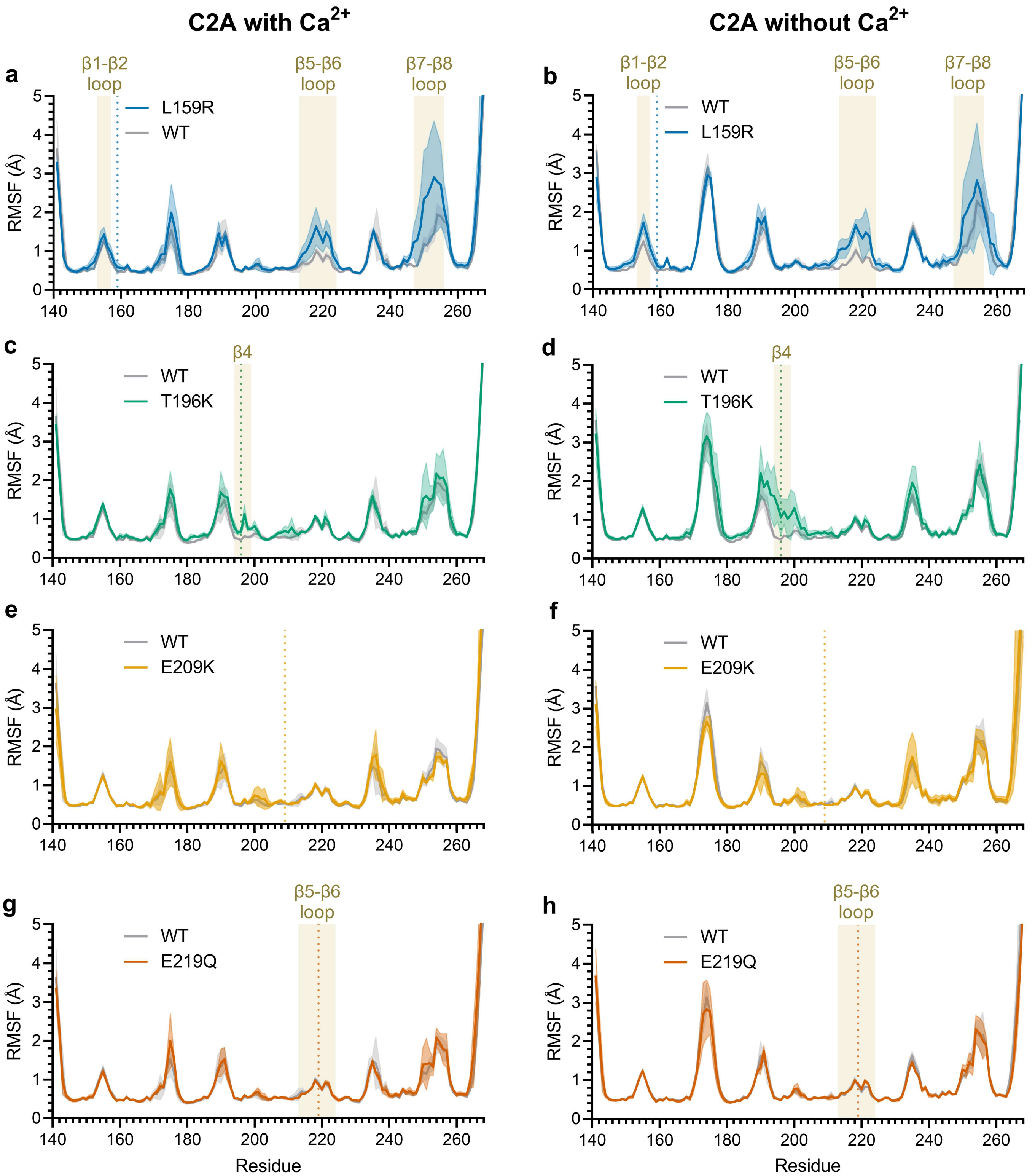

Supplementary Figure 3. Mean RMSF traces of C2A variants. Models of WT and variant SYT1 C2A domains, with Ca^{2+} ions either present (left) or removed (right), each underwent four ~400ns molecular dynamics simulations. RMSF (root-mean-square fluctuations) of the backbone C-alpha atoms of each novel variant (Leu159Arg (**a,b**), Thr196Lys (**c,d**), Glu209Lys (**e,f**), Glu219Gln (**g,h**)) and WT domains were measured over the course of the simulation and plotted for each residue. Shaded regions highlight specific features of the domain as labelled (β-strands and loops between β-strands). Vertical dotted lines indicate site of residue altered by variant. Data are mean ± SD of four simulations.

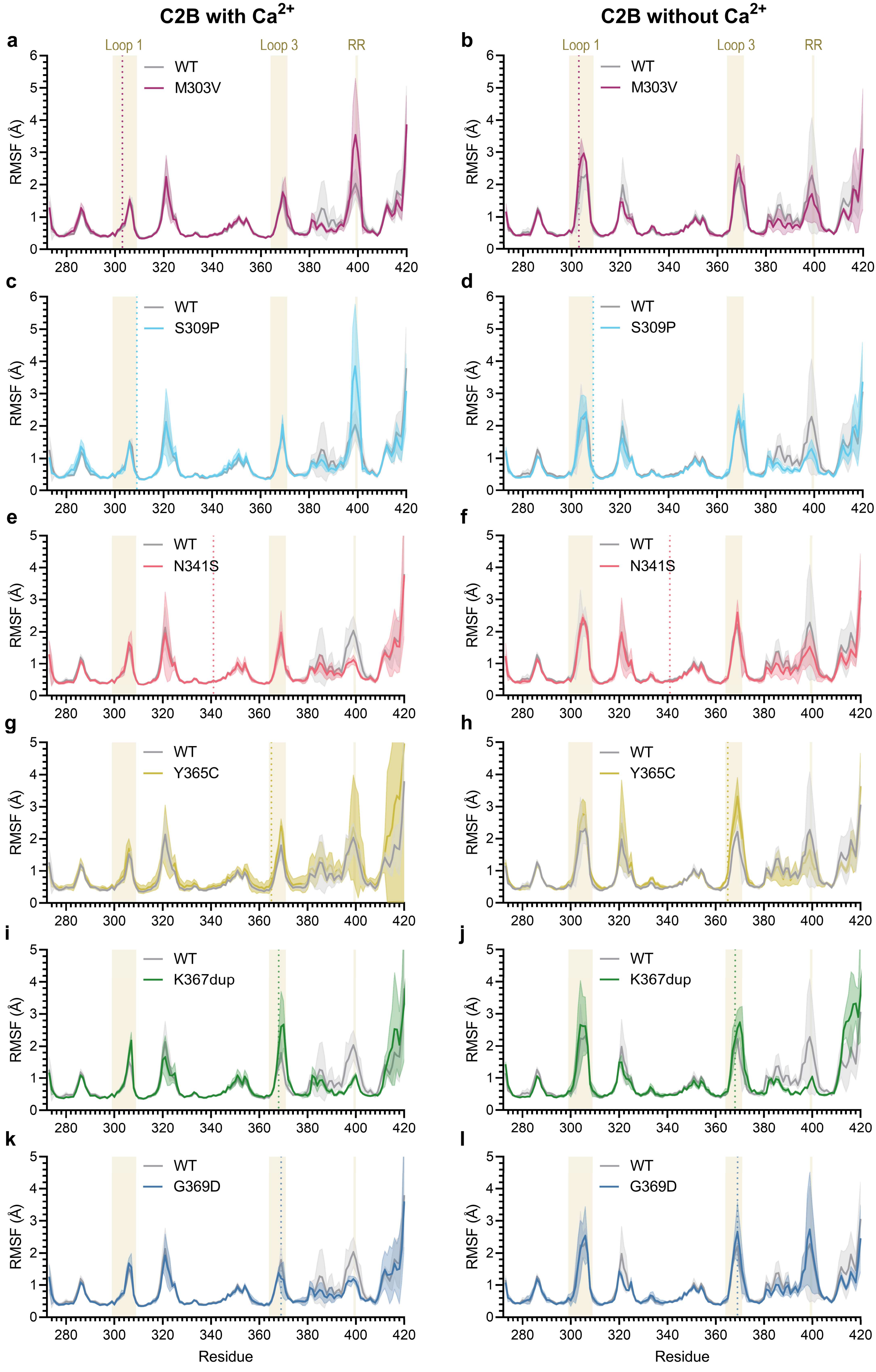

C2B with Ca2+
C2B without Ca2+
a
b
c
d
e
f
g
h
i
j
k
l
Loop 1
Loop 3
RR
WT
M303V
S309P
N341S
Y365C
K367dup
G369D
RMSF (Å)
Residue

Supplementary Figure 4. Mean RMSF traces of C2B variants. Models of WT and variant SYT1 C2B domains, with Ca^{2+} ions either present (left) or removed (right), each underwent four ~400ns molecular dynamics simulations. RMSF (root-mean-square fluctuations) of the backbone C-alpha atoms of each novel variant (Met303Val (**a,b**), Ser309Pro (**c,d**), Asn341Ser (**e,f**), Tyr365Cys (**g,h**), Lys367dup (**i,j**), Gly369Asp (**k,l**)) and WT domains were measured over the course of the simulation and plotted for each residue. Shaded regions highlight specific features of the domain as labelled (Ca^{2+}-binding loops 1 and 3, and arginine apex (RR) i.e. Asp399, Asp400). Vertical dotted lines indicate site of residue altered by variant. Data are mean ± SD of four simulations.

Variant	Donor	Acceptor	Bond Type	WT with Ca^{2+} Avg	WT with Ca^{2+} SD	Variant with Ca^{2+} Avg	Variant with Ca^{2+} SD	WT no Ca^{2+} Avg	WT no Ca^{2+} SD	Variant no Ca^{2+} Avg	Variant no Ca^{2+} SD
**E209K**	K197 backbone	E209 side chain	H-bond	37%	6%	-	-	14%	7%	-	-
	K197 side chain	E209 side chain	Salt Bridge	14%	2%	-	-	16%	2%	-	-
**E219Q**	K223 side chain	E219 side chain	Salt Bridge	22%	2%	1%	0%	25%	3%	1%	0%
	K223 side chain	E/Q219 backbone	H-bond	13%	4%	10%	2%	12%	2%	11%	2%
	E/Q219 backbone	P216 backbone	H-bond	25%	3%	15%	2%	25%	2%	18%	3%
**S309P**	T335 backbone	S/P309 backbone	H-bond	30%	2%	22%	2%	33%	2%	17%	3%
	S309 side chain	D304 side chain	H-bond	26%	18%	-	-	-	-	-	-
	M303 backbone	S309 side chain	H-bond	19%	9%	-	-	5%	2%	-	-
	S309 side chain	D364 side chain	H-bond	-	-	-	-	12%	15%	-	-

Supplementary Table 4. Select intramolecular bonds altered by SYT1 variants in molecular dynamics simulations. Values are percentage of frames in which this interaction is present, averaged across four trajectories. All amino acid numbering follows human sequence for simplicity, but note that "variant" simulations were performed using homology models derived from rat C2A and C2B structures (rat sequence is human sequence -1). Only hydrogen bonds present in at least 10% of frames of a simulation trajectory are shown.

Supplementary Table 5. Sample characteristics for questionnaire analysis.

		SYT1 group (n=14)	ID comparison group (n=51)	Statistical comparisons
Age[c]	*Mean (SD)*	10.5 (5.5)	12.59 (5.1)	*U*=456 *p*=0.11
	Range	4.4-25.8	4.4-25.7	
Sex	*% female (n)*	43 (6)	63 (32)	χ(1) = 1.79 *p* =0.18
Global adaptive function (Vineland Adaptive Behaviour Composite)	*Mean (SD)*	45.1 (18.6)	52.4 (13.4)	t=1.37 *p*=0.19
	Range	20-74	20-74	
Behavioural and emotional difficulties (DBC total problems T-score)[a,c]	*Mean*	55.9 (8.4)	57.2 (14.2)	*U*=262 *p*=0.84
	Range	40-68	38-106	
Social communication impairment (SRS total T-score)[b,d]	*Mean (SD)*	78.5 (11.6)	79.5 (10.2)	*T=0.24* *p*=0.81
	Range	57-93	58-98	

[a] Data available for all SYT1 group members and n=42 ID comparison group members
[b] Data available for n=11 SYT1 group members and n=40 ID comparison group members
[c] Independent-samples Mann-Whitney U test, asymptotic 2-sided significance
[d] Independent-samples t-test, 2-tailed significance, equal variances not assumed

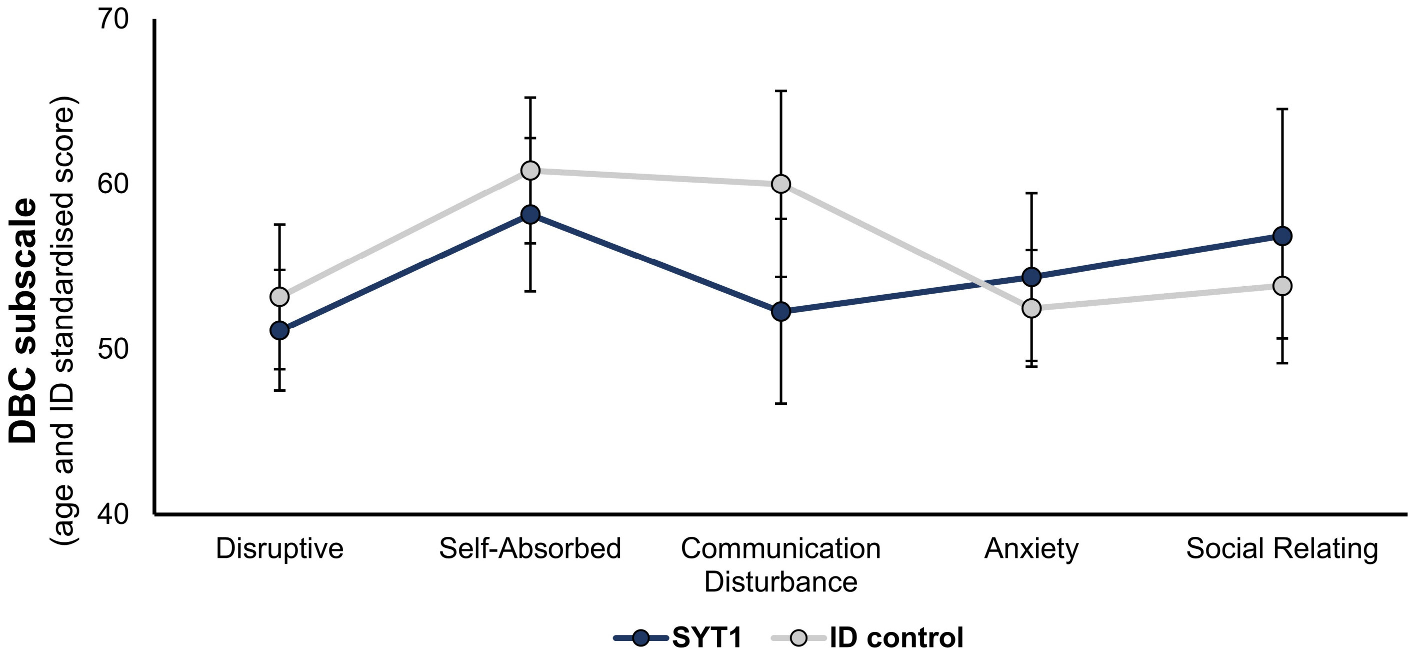

Supplementary Figure 5. Profile of behavioural and emotional problems in SYT1 cohort. Mean scores for each subscale of the DBC (Developmental Behaviour Checklist), standardised for age and global ability, are shown for SYT1 (n=14) and ID comparison (n=42) groups. T-scores ≥ 50 indicates impairments of likely clinical concern. Error bars represent 95% confidence intervals. No significant differences between groups were observed through general linear model analysis.

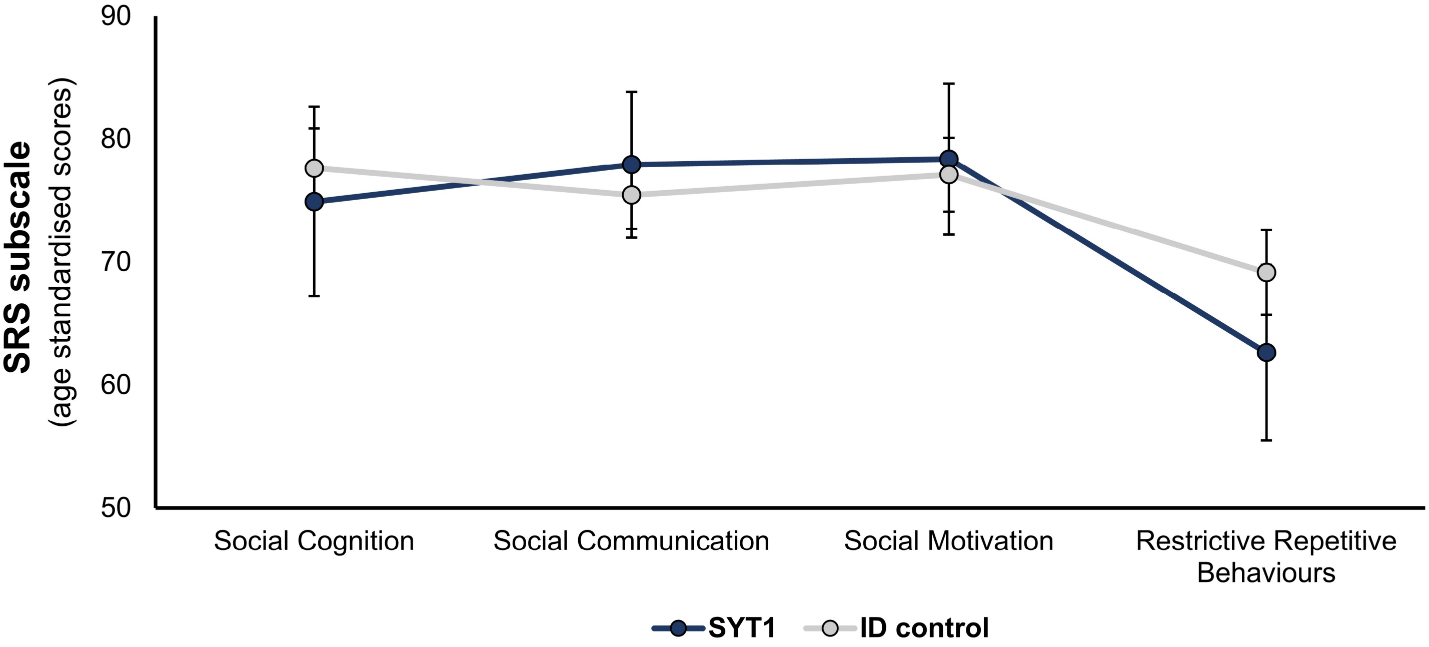

Supplementary Figure 6. Profile of autism-related social functions in SYT1 cohort. Mean scores for each subscale of the SRS (Social Responsiveness Scale) are shown for SYT1 (n=11) and ID comparison (n=40) groups. T-score ≥ 75 indicates impairments that are suggestive of possible autism diagnosis. Error bars represent 95% confidence intervals. No significant differences between groups were observed through general linear model analysis.

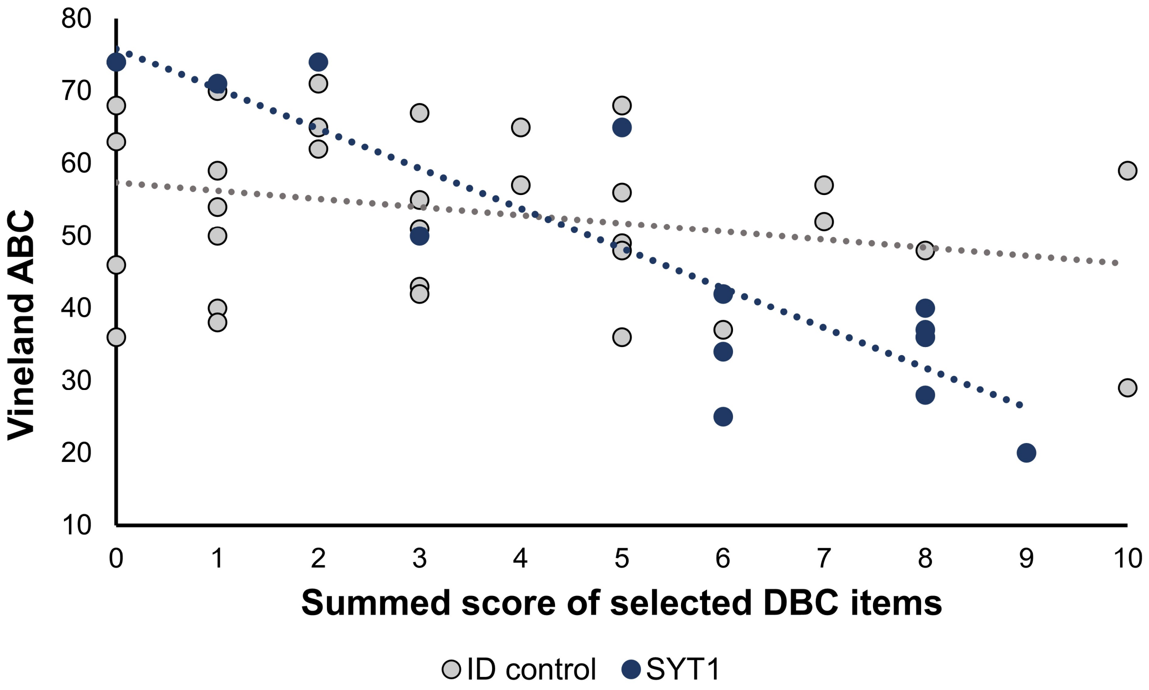

Supplementary Figure 7. Relationship between selected DBC items and Vineland ABC in SYT1 cohort. Five items relating to self-injury, mood instability and repetitive movements were selected from the DBC-P. Raw scores (0-2) for each item were summed for each participant. Significantly higher scores on these selected items were found in the SYT1 group (n=14) compared to ID controls (n=42; p=0.02). Data show these summed scores from selected DBC items plotted against the Vineland ABC score of each individual, with each data point representing one participant. Dotted lines show linear correlation between score for selected DBC items and Vineland ABC. A significant relationship between these scores was observed only in the SYT1 group (SYT1: Spearman's rho -0.73, p=0.003; ID controls: Spearman's rho -0.18, p=0.26; Fisher's test z=-2.19, p=0.01).

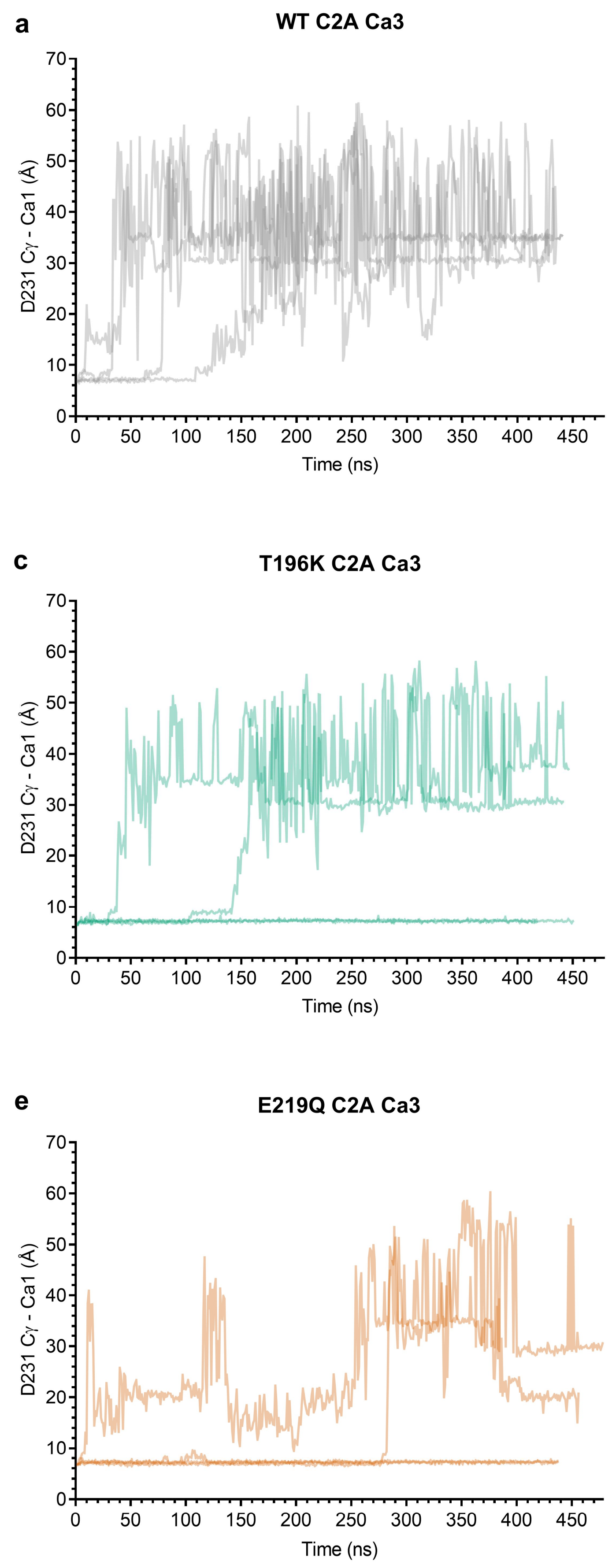

Supplementary Figure 8. Unstable binding of Ca3 in WT and variant C2A domains. Ca^{2+}-bound models of WT and variant SYT1 C2A domains each underwent four ~400ns molecular dynamics simulations. Retention of bound Ca^{2+} ions was assessed by measuring the distance between each Ca^{2+} atom and the Cγ of Asp231 of C2A in each simulation frame. Data show these distances over simulation time for each of the four trajectories per variant. (**a**) WT. (**b**) Leu159Arg. (**c**) Thr196Lys. (**d**) Glu209Lys. (**e**) Glu219Gln.

Supplementary References

1. Richards S, Aziz N, Bale S, et al. Standards and guidelines for the interpretation of sequence variants: a joint consensus recommendation of the American College of Medical Genetics and Genomics and the Association for Molecular Pathology. Genet Med. 2015;17(5):405-424.
2. Shao X, Fernandez I, Sudhof TC, Rizo J. Solution structures of the Ca2+-free and Ca2+-bound C2A domain of synaptotagmin I: does Ca2+ induce a conformational change? Biochemistry. 1998;37(46):16106-16115.
3. Fernandez I, Arac D, Ubach J, et al. Three-dimensional structure of the synaptotagmin 1 C2B-domain: synaptotagmin 1 as a phospholipid binding machine. Neuron. 2001;32(6):1057-1069.
4. Waterhouse A, Bertoni M, Bienert S, et al. SWISS-MODEL: homology modelling of protein structures and complexes. Nucleic Acids Research. 2018;46(W1):W296-W303.
5. Abraham MJ, Murtola T, Schulz R, et al. GROMACS: High performance molecular simulations through multi-level parallelism from laptops to supercomputers. SoftwareX. 2015;1–2:19-25.
6. Lindorff-Larsen K, Piana S, Palmo K, et al. Improved side-chain torsion potentials for the Amber ff99SB protein force field. Proteins. 2010;78(8):1950-1958.
7. Berendsen HJC, Postma JPM, van Gunsteren WF, Hermans J. Interaction Models for Water in Relation to Protein Hydration. In: Pullman B, editor. Intermolecular Forces: Proceedings of the Fourteenth Jerusalem Symposium on Quantum Chemistry and Biochemistry Held in Jerusalem, Israel, April 13–16, 1981. Dordrecht: Springer Netherlands; 1981:331-342.
8. Darden T, York D, Pedersen L. Particle mesh Ewald: An N·log(N) method for Ewald sums in large systems. The Journal of Chemical Physics. 1993;98(12):10089-10092.
9. Baker K, Gordon SL, Grozeva D, et al. Identification of a human synaptotagmin-1 mutation that perturbs synaptic vesicle cycling. J Clin Invest. 2015;125(4):1670-1678.
10. Berendsen HJC, Postma JPM, Gunsteren WFv, DiNola A, Haak JR. Molecular dynamics with coupling to an external bath. The Journal of Chemical Physics. 1984;81(8):3684-3690.
11. Bussi G, Donadio D, Parrinello M. Canonical sampling through velocity rescaling. J Chem Phys. 2007;126(1):014101.
12. Parrinello M, Rahman A. Polymorphic transitions in single crystals: A new molecular dynamics method. Journal of Applied Physics. 1981;52(12):7182-7190.
13. Baker K, Gordon SL, Melland H, et al. SYT1-associated neurodevelopmental disorder: a case series. Brain. 2018;141(9):2576-2591.
14. Ubach J, Zhang X, Shao X, Sudhof TC, Rizo J. Ca2+ binding to synaptotagmin: how many Ca2+ ions bind to the tip of a C2-domain? EMBO J. 1998;17(14):3921-3930.
15. Fernandez-Chacon R, Konigstorfer A, Gerber SH, et al. Synaptotagmin I functions as a calcium regulator of release probability. Nature. 2001;410(6824):41-49.
16. Kaplanis J, Samocha KE, Wiel L, et al. Evidence for 28 genetic disorders discovered by combining healthcare and research data. Nature. 2020;586(7831):757-762.
17. Cafiero C, Marangi G, Orteschi D, et al. Novel de novo heterozygous loss-of-function variants in MED13L and further delineation of the MED13L haploinsufficiency syndrome. Eur J Hum Genet. 2015;23(11):1499-1504.
18. Bradberry MM, Courtney NA, Dominguez MJ, et al. Molecular Basis for Synaptotagmin-1-Associated Neurodevelopmental Disorder. Neuron. 2020;107(1):52-64 e57.
