# Supplementary material for: Expanding the genotype and phenotype spectrum of SYT1-associated neurodevelopmental disorder
Source: Genet Med. 2022 Apr;24(4):880–93. doi: 10.1016/j.gim.2021.12.002 (PMC8986325; doi:10.1016/j.gim.2021.12.002)
Supplement: Supplemental Table 2 References [file mmc3.docx]

| **Supplemental References Table S2** |
| --- |
| 1. Kaplanis J, Samocha KE, Wiel L, et al. Evidence for 28 genetic disorders discovered by combining healthcare and research data. Nature. 2020;586(7831):757-762. |
| 2. Cafiero C, Marangi G, Orteschi D, et al. Novel de novo heterozygous loss-of-function variants in MED13L and further delineation of the MED13L haploinsufficiency syndrome. Eur J Hum Genet. 2015;23(11):1499-1504. |
| 3. Baker K, Gordon SL, Melland H, et al. SYT1-associated neurodevelopmental disorder: a case series. Brain. 2018;141(9):2576-2591. |
| 4. Bradberry MM, Courtney NA, Dominguez MJ, et al. Molecular Basis for Synaptotagmin-1-Associated Neurodevelopmental Disorder. Neuron. 2020;107(1):52-64 e57. |
| 5. Baker K, Gordon SL, Grozeva D, et al. Identification of a human synaptotagmin-1 mutation that perturbs synaptic vesicle cycling. J Clin Invest. 2015;125(4):1670-1678. |
